# Functional Studies of Plant Latex as a Rich Source of Bioactive Compounds: Focus on Proteins and Alkaloids

**DOI:** 10.3390/ijms222212427

**Published:** 2021-11-17

**Authors:** Joanna Gracz-Bernaciak, Oliwia Mazur, Robert Nawrot

**Affiliations:** Molecular Virology Research Unit, Institute of Experimental Biology, Faculty of Biology, Adam Mickiewicz University, Poznań, Uniwersytetu Poznańskiego 6, 61-614 Poznań, Poland; joanna.gracz-bernaciak@amu.edu.pl (J.G.-B.); oliwia.mazur@amu.edu.pl (O.M.)

**Keywords:** latex, antiviral proteins, antimicrobial compounds, cytotoxicity, drug discovery, *Chelidonium majus*, CRISPR/Cas9

## Abstract

Latex, a sticky emulsion produced by specialized cells called laticifers, is a crucial part of a plant’s defense system against herbivory and pathogens. It consists of a broad spectrum of active compounds, which are beneficial not only for plants, but for human health as well, enough to mention the use of morphine or codeine from poppy latex. Here, we reviewed latex’s general role in plant physiology and the significance of particular compounds (alkaloids and proteins) to its defense system with the example of *Chelidonium majus* L. from the poppy family. We further attempt to present latex chemicals used so far in medicine and then focus on functional studies of proteins and other compounds with potential pharmacological activities using modern techniques such as CRISPR/Cas9 gene editing. Despite the centuries-old tradition of using latex-bearing plants in therapies, there are still a lot of promising molecules waiting to be explored.

## 1. Introduction

Latex-bearing plants have a long history of benefiting human health and medicinal use in many different regions and cultures all over the world. Recent research suggests that the opium poppy (*Papaver somniferum* L.) was already in the process of domestication at the end of 4th millennium BC [1] and early domesticated ancestors of *Cannabis sativa* L. diverged ~10,000 years BC [2]. Those are two leading examples of laticiferous plant species used for therapies and together with *Hevea brasiliensis* Muli. Arg., which is the main and irreplaceable natural rubber source, have the best known and described latex composition. These complex fluids consist of different secondary metabolites, like terpenes, alkaloids, or phenolics, and jointly with a broad range of proteins are the first line of plant herbivore defense system. Another extensively studied laticiferous medicinal plant is Greater Celandine (*Chelidonium majus* L.), a relative of the opium poppy, which is a rich source of numerous biologically active compounds, used in traditional folk medicine as antiviral, antibacterial, antifungal, choleretic, and anticancer agents [3,4,5,6]. Many compounds of latex are active in both eukaryotic and prokaryotic organisms [7]. At present, when mankind is running out of antibiotics and other antimicrobial compounds, new cancer therapies are still needed, and to make matters worse, the scale of pest and microbial resistance is increasing. Thus, the exploration of such rich natural deposits of active molecules is a very promising research direction.

This review paper characterizes the main components of latex and summarizes their known potentially therapeutic activities. We focus on two classes of compounds, proteins and alkaloids, which represent complex macromolecules and low-molecular compounds. Both types of molecules co-exist in plant latex and possibly actively cooperate in a synergistic manner to enable and boost their biological activities. We propose a model of antiviral latex activity and present examples of CRISPR/Cas9 editing genomes, which can shed light on a complex network of specialized metabolites synthesis and interactions or complementation. Despite its long history of use, there is still room for improvement of agronomic traits in domesticated latex-bearing plants and for the exploitation of wild species latex compounds to prepare a range of novel compounds of therapeutic potential, as well as novel drugs and drug carriers.

## 2. Diversity and Role of Latex in Plant Physiology

Latex is a milky emulsion produced by complex secretory structures called laticifers. It is defined as a suspension of various particles (organic and inorganic) dispersed in a liquid with different refractive index. Depending on prevalent content and plant species studied, it can be milky white or yellowish, orange to brown or even colorless. However, it is more than a liquid. It is identified as a laticifer’s protoplast with mitochondria, plastids, endoplasmic reticulum, Golgi bodies, polyribosomes, and vacuoles [8]. Laticifers are latex-producing, highly specialized plant cells or connected cells, which are spread through the whole plant body in the form of linear tubes which can grow and elongate with plant organs. They can be found in almost every part of latescent plants, namely in the root, stem, leaf, sepal, petal, stamen, ovary, and stigma, or they can occur only in some particular organs. Latex occupies the whole volume of the laticifer system [9].

Latex was identified in at least 20,000 plant species belonging to 43 families of vascular plants. Most of them are Angiosperms (41 families), one family belongs to ferns, and one to gymnosperms [8,9,10]. In a great example of convergent evolution events in the plant kingdom, latex occurrences take place several times in phylogenetically unrelated orders. Laticifers developed in both monocotyledonous and dicotyledonous, in the basal clades (Ana-grade), magnoliids, monocots, basal eudicots, rosids, and asterides [11,12]. One morphotype of laticifers, articulated, which are fused chains of cells with intact, porous, or even absent terminal walls, form laticiferous vessels and were recorded in 27 families. The other main morphotype is called non-articulated, which occurs more rarely and is formed by a single plant cell with almost infinite growth potential. Both types of laticifers can extend not only longitudinally with the growth of organs, but also radically create branched networks of tubes. For the proper classification of the laticifer system, it is essential to use plant material with embryos or meristems. Only based on ontology can articulated and non-articulated morphotypes be distinguished (analysis of number of precursor cells and phase of laticifers development often coupled with analysis of laticifers enzymatic activity of pectinases and chitinases, with the latter active only in articulated laticifers [13]). Previously, cases of incorrect assignment to the appropriate laticifer types were described, e.g., for mulberry [14,15] or for *Ficus montana* Burm.f. and *Maclura tinctoria* L. [13]. Attempts were made to use types of laticifers as a diagnostic tool for some taxa, but it is more likely that different morphotypes will be found in different species within the same family [11].

Nowadays, it is well established that the biological role of latex is plant defense against herbivores and pathogens [10], but in 1989 Webster and Baulk concluded that the function of latex was unknown [16]. Many latex metabolites are stored within large vacuoles and are released after being physically damaged at the site of injury. Some of them act as toxic and dissuasive components. After mechanical disruption of plant tissue, latex is immediately released and is the first line of plant defense. Thanks to its inherent stickiness and coagulation properties, latex forms a barrier against pathogen invasion. Moreover, latex’s rapid coagulation and high viscosity can restrict herbivore movements, as well as immobilize mouthparts and other sense organs [17]. This strategy gives an advantage to latescent plants, especially in environments with high a herbivory rate, like tropical or subtropical forests [18].

## 3. Main Components of Latex-Secondary Metabolites

Taking into consideration the defense role of latex in plant development, it should not be surprising how complex and diverse the latex composition can be. Despite tremendous variability in latex components, which is dependent on the species, phase of development, external and internal stimuli, and stresses [19], two major groups of biologically active compounds can be distinguished, namely secondary metabolites and proteins. Many of those products are cytotoxic and it was suggested that laticifers evolved as sequestering compartments, which ensure the storage of such substances regardless of the vascular system. This solution provides a unique and preformed defense mechanism with almost immediate response to herbivory attack. The internal pressure of latex causes the secretion of concentrated active substances at the point of damage in a few seconds. In contrast, an inducible defense system needs hours or even days to synthesize and collect sufficient amounts of active substances to act against pathogens [20]. In the context of latex composition, it is worth noticing that a synergetic mode of action was established for some of its constituents, like terpenes associated with phenolic compounds [20,21] or different proteins exhibiting defense functions against insects or fungi [22,23]. Therapeutic properties of selected latex compounds are described in detail in Section 5. Below, we present a short characterization of secondary metabolites common for latex-bearing plants.

Secondary metabolites are a heterogeneous group of chemical compounds not essential to vegetative growth, but for plant adaptation to changes in the external as well as internal environment. As mentioned before, the presence of specific metabolites in latex is a highly species-specific trait, but in general terpenes, phenolics, alkaloids, and cardenolides are present in most of the laticifer types.

One of the most abundant groups of secondary metabolites in plant latex are isoprene-derived compounds, terpenes. Within this group, the most economically important member is rubber (cis-1,4-polyisoprene), which is found in 2500 plant species (300 plant genera from eight families) [7,24], but harvested on global scale from one, *Hevea brasiliensis* species. Rubber particles may constitute up to 50% of *H. brasiliensis* latex volume [25]. It is proposed that the main function of rubber is related to the coagulation process [20], but it was also suggested that rubber biosynthesis accumulate excess of photosynthate and prevent damage to photosynthesis apparatus under stress conditions [26].

Apart from rubber, in latex of *Euphorbia* genus, some triterpenoids (i.e., cycloartenol, 24-methylenecycloartenol, lupeol, lupeol acetate, lanosterol, and 24-methylenelanosterol) are always present in higher concentration than in other organs. Similarly, latex of *Lactuca sativa* L. contains high levels of several sesquiterpene lactones (concentration of lactucopicrin oxalate is 1000 higher than in leaves) [27]. Those terpenoids are often accompanied with steroids. It is presumed that together they can disrupt cell walls of insects or microorganisms, intercalate membranes, and form channels which enable the migration of small, toxic, hydrophilic molecules such as phenolics inside the cell [28].

Phenolics (e.g., tannins, lignans, coumarins, flavonoids), another broad group of secondary metabolites found in latex, have been known for their antioxidant properties and taking part in response to oxidative stress conditions [29]. They are mainly products of shikimate pathways and were found, among others, in latex of sweet potato *Ipomoea batatas* L. The overall concentration of p-coumarate esters exceeded 3% fresh vine latex and 10% root latex of the variety “Jewel”. The presence of those phenolics is inversely correlated with the acceptability of sweet potato by weevils [30], which supports the anti-herbivore latex function. Different polyphenol compounds were identified in latex of *H. brasiliensis*, namely gallic acid, naphthoic acid, quercetin, chlorogenic acid, and rutin, which are also postulated to play a role in plant defense system [31]. In common dandelion (*Taraxacum officinale* F.H. Wigg.), phenolic esters, next to sesquiterpene lactones and triterpene acetates, were found in high concentration in the main root. Those active compounds showed a repelling effect in experiments with *Diabrotica balteata* larvae [32].

**Figure 1:**
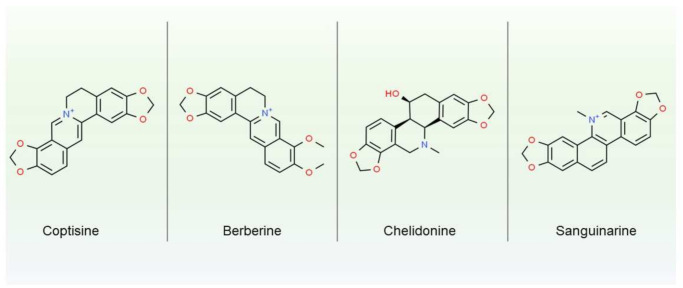
Chemical structure of four most studied alkaloids from *Chelidonium majus*.

Another group of secondary metabolites sequestered in laticifers are alkaloids. Those amino acid derivatives, which are highly bioactive and often toxic, serve eco-physiological functions in plants, providing better fitness to specific environmental niches [33]. Alkaloids for thousands of years have been used and abused by humans, even leading to military conflicts (like opium wars in the 19th century or ongoing drug wars in many countries). Those low molecular compounds were found amongst 35 families, mostly angiosperms, including Apocynaceae, Papaveraceae, and Moracea [8]. The best known and described example of laticifers rich in alkaloids is opium, namely the dried latex of *P. somniferum* used in folk and traditional medicine, as well as psychedelic drugs. Opium contains at least 20 alkaloids, such as morphine, papaverine, and codeine. Morphine may constitute up to 5% of fresh latex, and codeine up to 1% [34]. Latex of *Chelidonium majus*, a species closely related to *P. somniferum*, is also a rich source of bioactive alkaloids, e.g., chelidonine, sanguinarine, berberine or coptisine (chemical structures are depicted on Figure 1). Those isoquinoline alkaloids can reach up to 20% of fresh latex mass [35] and are known for their multiple pharmacological effects (antioxidant, anti-inflammatory, anticancer, anti-neurodegenerative, and antimicrobial). Although *C. majus* is related to *P. somniferum*, its latex does not contain morphine-like alkaloids, such as morphine or codeine, and therefore does not have sedative effects. Yet, some of the *C. majus* secondary metabolites, like berberine and chelidonine, as well as protein enriched extracts can have analgesic effects, similar to morphine [36]

Cardenolides, a specific type of steroids (cardiac glycoside) consisting of sugar, steroid, and lactone, are the next group of metabolites commonly occurring in latex. There is no known function of those compounds, other than defense. Cardenolides are inhibitors of Na^+^/K^+^-ATPases, which are essential to maintain cell electrical potential, regulate cellular volume, and take part in transmembrane transport. As inhibitors cardiac glycosides are remarkably toxic to most animals, therefore latex of *Antiaris toxicaria* Lesch. (Moraceae) rich in cardenolides, were used by South Asia tribes as a poison on darts during hunting [37]. It is worth mentioning the widely known interaction between cardenolides from *Asclepias* spp. and monarch butterflies (*Danaus plexippus*). Monarch larvae, which feed on plants containing cardenolides, have developed the ability to sequestrate those compounds, which then cumulate in wings and protect them from bird predators [38,39].

## 4. Spectrum of Latex Proteins

Laticifers are not only a reservoir system for low-molecular weight defense compounds, but as mentioned earlier, constitute a living cell, and as one, has a distinct proteome. A wide range of both constitutive and inducible proteins are present in latex, with huge diversity between different plant species. Comprehensive studies of 1208 latex proteins from 20 various latex-bearing plants led to the identification of 887 non-redundant proteins from three main species, i.e., *L. sativa*, *H. brasiliensis,* and *P. somniferum*. Only 11 proteins were found in all three species [40], which represents well-illustrated variability within latex proteomes. In a set of 887 proteins, GO enrichment analysis showed that response to chemical and abiotic stimuli categories were highly overrepresented. Within this category, at least 21 proteins are involved in response to cadmium ion stress, while another 13 proteins were associated with response to high salt conditions. Proteins with function in defense were also identified. Amongst them two proteins, glycosyl hydrolase superfamily proteins (AT4G16260) and the NAD(P)H subunit NDH-N (AT5G58260), were pinpointed for their response to fungi infections. In the category of cellular components, most proteins were located in cytoplasm, but terms related to intracellular membrane-bounded organelles (including plastids and mitochondria) were highly enriched as well. Cho and colleagues also compared latex and phloem proteomes. GO terms common for those two conductive systems were related to metabolic pathways like the metabolism of nitrogen compounds, amine, alcohol, hexose, carboxylic acid, and carbohydrate catabolism. Stress response related terms were shared in laticifers and phloem systems, but for example, osmotic stress GO terms were identified only in latex. On the other hand, GO terms connected with response to zinc were found only in phloem [40]. Despite some similarities with functionally related conduit systems, laticifers present a unique and distinct set of proteins. Nevertheless, we can distinguish some common protein functional groups prevalent in different laticiferous plant species, like proteases, protease inhibitors, lectins, oxidases, chitinases, or defense-related proteins. Below, we survey those common for latex protein’s groups.

The most frequently reported latex proteins are proteases. One of the best described examples of protease in laticiferous plants is papain, a cysteine protease in latex of the Papaya tree (*Carica papaya* L.). Papain is an enzyme practically used as a meat tenderizer and in the cosmetic industry. In papaya latex, its concentration is 200 times higher than in leaf tissue, which leads to 20 times higher papain activity [41]. Experiments with protease inhibitors, E-64 and larvae of Eri silkworm, give clear evidence of papain involvement in plant resistance against herbivory (leaf toxicity to Eri silkworm was lost after covering its surface with E-64). Similarly, ficin (cysteine protease from fig) inhibition by E-64 makes fig leaf edible for insects [41]. Nonetheless, the exact site of action and mechanism of protease toxicity remains unclear. Other groups of proteases-serine proteases-were found in Moraceae, Euphorbiaceae, Apocynaceae, and Convolvulaceae families. Rarely are those two protease classes (cysteine and serine) identified in the latex of the same species. More often, one type of peptidases is described for a particular plant. Known exceptions are species from the *Plumeria* genus, where both proteolytic mechanisms have been reported [42].

In the latex of many plants, not only were proteases identified, but protease inhibitors as well. Those compounds bind to proteases and prevent the digestion of proteins, thus causing a shortage of amino acids and impairing the growth and development of aggressors [8]. Protease inhibitors belong to group 6 of Pathogenesis-Related (PR) proteins and their role in plant defense in species without latex is well established [43]. In laticiferous species *Ficus carica* L., the expression level of trypsin inhibitor increases significantly after wounding and jasmonic acid treatment [43,44]. Moreover, in papaya latex, trypsin inhibitor (together with class-II chitinase and a glutaminyl cyclase) was one of the compounds accumulated after mechanical damage [45].

Lectins are proteins with one or more domains which enable them to recognize and bind to specific sugar structure (in free form or as a part of glycoproteins and glycolipids). They are compounds necessary for the perception of possible invasion by recognizing specific glycans at damage sites and from pathogens. Several types of lectins were identified in the families Euphorbiaceae, Moraceae and Apocynaceae. Hevein, the major latex protein from *H. brasiliensis*, is responsible for rubber agglutination, by bridging rubber particles after the recognition of 22 kDa glycoprotein receptor [46]. This small 43 amino acid protein with lectin domain turned out to be the main contact allergen from natural rubber [46,47]. In contrast to indirect mechanisms of latex coagulation, there are studies reporting a straight negative impact of lectins on pests from different families, e.g., Lepidoptera, Coleoptera, Diptera and Hemiptera [18]. It was shown that the expression of exogenous lectins in genetically engineered plants led to various detrimental effects in invading insects, ranging from a severe delay in development to high mortality rates. Moreover, the introduction of specific lectins to the insect diet negatively affects pest performance, as reviewed in [48]. The toxicity of lectins depends on the presence of specific carbohydrates in the insect body, which, in turn, is correlated with insects’ developmental stage [49,50].

Latex from plants belonging to families Euphorbiaceae, Moraceae, and Anacardiaceae shows oxidase activity [51]. Polyphenol oxidase (PPO) and peroxidase (POD) are the most commonly occurring peptides. Both enzymes are known for their role in plant defense, not only in lactiferous plant species. Polyphenol oxidase catalyzes the oxidation of phenols to o-quinones, which are highly reactive and secondary non-enzymatic reactions lead to formation of polymers with protein functional groups. At least three mechanisms of PPO toxicity to insects were proposed: decreased nutritive value of leaf proteins, oxidative burst in insect gut, and direct toxicity of PPO catalyzed products [52]. Products of PPO activity and polymerization are black, brown, or red in color and are responsible for the darkening of latex after contact with air. Peroxidases on the other hand are H_2_O_2_ scavengers–they catalyze oxidation of phenolic substrates using H_2_O_2_ as an electron acceptor. Subsequent reactions lead to cross-linking products of phenolic compounds, such as lignin or suberin [52,53]. The ability to reduce ROS makes those enzymes a crucial part of the antioxidant system, which is often triggered in response to both biotic and abiotic stress conditions. As a downstream result of peroxidase activity, the lignification of the plant cell walls occurs, which inhibits, for example, heavy metal entry [54].

Chitinases hydrolyze the β-1,4-glycosidic bonds of chitin, which builds fungi cell walls and insects exoskeletons and peritrophic matrix. Chitinases are classified as pathogenesis related proteins (PR proteins) and are expressed inducible or constitutively in tissues vulnerable to pathogen attack, like laticifers. Several ways of direct chitinase involvement in antifungal defense were established. Plant chitinases can inhibit hyphal growth. The overexpression of those enzymes in transgenic plants increased pathogen resistance in vivo. Meanwhile, products of chitin breakdown, via phytoalexins action, to induce systemic defense response [55]. Insecticidal effects constitute another well documented chitinase activity. For example, after the addition of two chitinases from mulberry (Morus sp.) to the *Drosophila melanogaster* larvae diet, 80% were found to be dead [56]. Chitinases identified from *Calotropis procera* (Aiton) W.T.Aiton affected larval survival and weight, mean developmental time and emergence of adults [23]. The mechanism of chitinase’s action on insects is not well established. It is postulated that it may involve the hydrolysis of chitin present in their peritrophic membranes, which are responsible for protection against mechanical damage and invasion by microorganisms or parasites. After its destruction, fatal infection can develop [57,58]. Moreover, in latex, chitinases present multiple isoforms. Three isoforms were purified and characterized from *Ficus microcarpa* latex [59], six basic chitinases were identified in *H. brasiliensis* latex [60] and at least 15 isoforms in latex of *C. procera* [61]. Different isoforms exhibit differences in sequences as well as in post-translational modification. Particular isoforms may in consequence differ in structural and biological properties, which need to be included in further studies regarding plant chitinases [61].

As mentioned before, chitinases belong to the group of pathogenesis related proteins. All PR proteins (as the name suggests) take part in plant defense mechanisms, but they are a very diverse group in the context of structures and activities. They were categorized into 17 distinct families (summarized in Table 1). For instance in *C. majus* latex representatives of 12 families were identified [62,63]. Two quantitatively predominant families were PR-9 (peroxidases described previously) and major latex proteins (MLPs), which are homologs of PR-10 proteins (ribonuclease-like/Bet v1 protein family). Similarly, in latex of *P. somniferum,* those two families were also overrepresented. In another well studied laticiferous plant, *H. brasiliensis*, members of 9 PR families were described [8]. PR proteins are low molecular weight and are induced by phytopathogens as well as defense-related signaling molecules, like salicylic and jasmonic acid. Due to their mode of action in biotic and abiotic stress conditions, they are one of the most promising targets for engineering multiple stress tolerant varieties [8,63].

One of the most interesting latex proteins is a major latex protein (MLP). It was discovered for the first time in the latex of the opium poppy (*P. somniferum*) in 1980′s by Nessler et al. Despite its abundance (up to 50% of soluble *P. somniferum* latex sub proteome) shown using SDS-PAGE and the presence of laticifer-specific peptides, its function remained unknown [64,65,66]. MLPs, being homologs of opium poppy major latex protein, have also been found in other non-latex-bearing plants, namely peach fruits (*Prunus persica* L. Batsch, cv Springcrest) [67], *Arabidopsis thaliana* L. [68], ripening kiwi fruit [69], *Panax ginseng* C.A. Meyer [70], tobacco (*Nicotiana tabacum* L.) [71], pepper (*Capsicum annuum* L.) [72], and cotton [73]. Recent data have shown the functional role of MLPs resembling the function of PR-10 proteins family [67], member of which are involved in the defense of plants against different pathogens and participate in plant metabolism [74,75,76]. The first proposed ribonuclease activity was demonstrated in major birch pollen allergen, Bet v1 which expresses homology to PR-10-like protein from white lupin (*Lupinus albus* L.) [77]. The most distinctive feature of Bet v1 protein superfamily (Pfam: PF00407) is a large solvent accessible hydrophobic cavity which might potentially function as a site to bind ligands [78]. Both MLPs and PR-10 families are members of Bet v1 superfamily although their sequence similarity is low and is below 25% [68]. Major latex protein/ripening-related proteins (MLP/RRP) subfamily is the second largest subfamily within the plant kingdom. It has 60 members, from which 31 are present in *A. thaliana*. The biological function of MLPs is still unknown, but there are assumptions that they are associated with development of fruit and flower along with response to stress and defense [78]. One of the recent studies has investigated the potential antiviral activity of MLP proteins against potato virus Y (PVY). It is transmitted by aphids and can cause mosaic, dwarfism, mottle, deformities, and even lead to necrosis in tobacco plants. MLP-like protein 28 (NbMLP28) from *Nicotiana benthamiana* Domin. was identified and cloned. Its expression profile has shown responsiveness towards PVY infection and defense-related signaling molecules, such as JA, SA, and ET. Virus-induced silencing of NbMLP28 made plants more susceptible to infection by PVY, though the transient overexpression of NbMLP28 gene improved resistance towards PVY in tobacco plants. The pathway responsible for modulation of the expression of NbMLP28 gene in *N. benthamiana* has also been identified. It showed the cis-acting elements in response to JA, light, auxin, drought, and endosperm expression to be present in the promoter sequence of NbMLP28 [79]. Antiviral properties towards TMV-P0 virus have been proven for CaPR10 proteins from hot pepper which have inhibited the viral penetration and/or replication. This study showed that CaPR10 after inoculation with the virus is phosphorylated and functions as RNase cleaving viral RNA [71]. During the studies on melon phloem-sap proteome, it was also found that major latex protein was present in the sap collected from the cucumber mosaic virus (CMV)-MP-expressing plants and CMV-infected plants [80]. Proteomic studies of *C. majus* latex have shown that MLP is highly overrepresented in *C. majus* latex [78] and can be seen at different stages of plant development till the fruit ripening. It is accumulated early in laticifer development and persists till maturity [78]. *C. majus* MLP (CmMLP) is composed of 147 amino acids, has a molecular weight of 16.77 kDa and theoretical pI 5.88, which corresponds to the typical sizes of MLPs (17 kDa) in other plants [74]. MLPs bind hormones and other metabolites with their conserved hydrophobic cavity during plant growth and development [81]. MLPs from other species, including *A. thaliana*, *N. benthamiana*, or *Cucumis melo* L., as mentioned previously, have also been proved to be involved in antiviral response. This can help to explain the potential antiviral activity of *C. majus* among its other biomedical properties [82] and could serve as a potential molecular target to be used in pharmacology or medicine and to improve the defense potential of agriculturally important crops against viral and non-viral pathogens.

**Table 1:** Summary of PR proteins previously identified in latex-bearing plants.

PR Proteins	Function	Latex-Bearing Plant Species	Reference
PR 2	β-1,3-glucanases	*Chelidonium majus* *Hevea brasiliensis*	[78,83]
PR 3	Class I, II, IV, V, VI, VII Chitinases	*Chelidonium majus* *Hevea brasiliensis*	[78,83]
PR 4	Class I, II Chitinases	*Chelidonium majus* *Hevea brasiliensis* *Carica papaya*	[45,78,83]
PR 5	Thaumatin-like proteins	*Chelidonium majus* *Hevea brasiliensis*	[78,83]
PR 6	Proteinase inhibitor	*Hevea brasiliensis* *Ficus carica* *Carica papaya*	[44,45,83]
PR 7	Endoproteinase	*Chelidonium majus* *Hevea brasiliensis*	[78,83]
PR 8	Class III Chitinase	*Hevea brasiliensis*	[83]
PR 9	Peroxidase	*Chelidonium majus* *Hevea brasiliensis* *Papaver somniferum*	[78,83,84]
PR 10	Ribonuclease-like proteins	*Chelidonium majus* *Papaver somniferum*	[78,84]
PR 11	Class I Chitinase	*Chelidonium majus*	[78]
PR 12	Defensin	*Chelidonium majus* *Hevea brasiliensis*	[78,83]
PR 14	Lipid-transfer protein	*Chelidonium majus* *Hevea brasiliensis*	[78,83]
PR 15	Oxalate oxidase	*Chelidonium majus*	[78]
PR 16	Oxidase-like	*Chelidonium majus*	[78]
PR 17	Antifungal and antiviral	*Chelidonium majus*	[78]

## 5. Biomedical Properties of Latex from Selected Plants with the Focus on *Chelidonium majus* L.

Nowadays, the demand for complementary therapeutics with herbal medicine including plant latex extracts, is constantly rising. Approximately 40% of drugs available at the market contain herbal active ingredients and the number is still growing [85]. Herbal medicines can act synergistically with currently used therapeutics and have fewer side effects compared to synthetic drugs. Both wild medical plants such as *C. majus* and domesticated species can serve as a source of raw materials (extracts) from which effective remedies can be obtained. *C. majus* is a plant that has been used for centuries to treat warts, papillae, and condylomas which are epidermal symptoms of human papillomavirus (HPV) infection [78]. In North America and Great Britain, it was used as a cure for infantile jaundice and ulcers of the eye. In traditional Chinese medicine, it was also used to fight fever, diminish the cough, promote diuresis in edema and ascites, alleviate the pain and treat blood stasis [86]. To date, it is still exploited in homeopathy and according to Foster et al. it can be used for liver and gallbladder disorders along with rheumatism and respiratory inflammations [87]. Although the molecular mechanism of *C. majus* latex compounds action is largely unknown, its antiparasitic, insecticidal, anti-neoplastic, antiproliferative, antimycotic, immunomodulating, and antiviral properties are well established [4,20,85,88,89,90].

*C. majus* belongs to the Papaveraceae family which also includes plants like bloodroot (*Sanguinaria canadensis* L.), persian poppy (*Papaver bracteatum* Lindl.), or opium poppy (*P. somniferum*). As mentioned before, *P. somniferum* serves as a source of several pharmacologically active substances, such as papaverine with vasodilator activity, noscapine, and sanguinarine with antimicrobial properties [85,91]. So far, it remains the only commercial source for benzylisoquinoline alkaloids, e.g., codeine, morphine, and its semi-synthetic forms like naltrexone or oxycodone, which are broadly used in medicine worldwide. *C. majus* is also a very important plant concerning its latex with a broad range of different components, connected with its multiple biological activities [3,4,5]. California poppy (*Eschscholzia californica* Cham.) is used for the production of bioactive compounds in vitro. Similarly to *P. somniferum,* it is involved in synthesis of benzylisoquinoline alkaloids like chelerythrine or sanguinarine [85]. Biomedical properties of different poppy species with the focus on *P. somniferum* have been already broadly studied and well described in the recent review article of Labanca et al. [92], and therefore it is not necessary to cover in the scope of this article. Another plant whose latex is rich in different molecules with biomedical properties including psychoactive ones is *Cannabis sativa*. It contains phytocannabinoids such as tetrahydrocannabinol (THC) and cannabidiol (CBD). THC has been shown to exhibit anti-cancer, analgesic, muscle relaxing, neuro-antioxidative, anti-inflammatory, and antispasmodic activity [93,94]. CBD also has numerous pharmacological properties and can be used in the case of metabolic syndrome, type I diabetic cardiomyopathy, or inflammatory lung diseases along with many others described in the review article of Burstein S. [95].

Nevertheless, the exact molecular mechanism responsible for many biomedical properties of laticiferous plant species is still not well established. Yet, the research community managed to define some interesting therapeutic activities and latex active compounds responsible for their occurrence. Those examples are presented below.

### 5.1. Antiviral Activity

In several assays, the ability of latex extracts was confirmed to act not only against plant viruses, but animal and human as well. As previously mentioned, *C. majus* has been effective against skin symptoms of HPV infections and different human viruses (HPV, HSV-1, HIV), although the mechanism of this activity is still undiscovered [96,97,98]. It is assumed that two groups of proteins are responsible for antiviral properties-peroxidases and nucleic acid binding proteins (see Table 2). Mechanical damage of the plant cell is accompanied by oxidative burst, which prevents any virus from the possible entry [99,100]. Second lines of defense include proteins such as lipoxygenases (LOX) and peroxidases (POX) which generate the H_2_O_2_ after pathogen attack [78,101]. The third line includes nucleic acid binding proteins, such as MLPs and/or GRPs, which have deoxyribonucleic and ribonucleic activities that allow them to potentially digest the viral RNA and DNA or act in yet not known mechanisms [74,78,102,103]. Another proposed mechanism is based on satellite RNA (satRNA) encapsulation and has been found in plants without significant symptoms of cucumber mosaic virus (CMV) infection. Primary plant defense is based on the silencing of RNA but PTI-based (pattern-triggered immunity-based) innate immune response is connected to antiviral defense. PTI is enabled by conserved pathogen-associated molecular patterns (PAMPs) detected by transmembrane pattern recognition receptors. On the other hand, effector-triggered immunity (ETI) is an “amplified” version of PTI, often associated with hypersensitive response (HR) and programmed cell death (PCD). In this mechanism, proteins from the NBS-LRR family (ETI-based R proteins) could recognize the avirulence (Avr) proteins of RNA viruses, effectors of non-viral origin, and trigger apoptosis in virus-resistant hosts. Avr proteins can function as silencing suppressors, which leads to the statement that innate immunity (both PTI and ETI) can be involved in the mechanism of the fight against plant viruses and can serve as a hint to understanding the activity against animal and human viruses [99,104]. Not only are proteins, but also certain alkaloids crucial to latex antiviral activity. Different secondary metabolites have already shown antiviral properties against herpes simplex virus and human adenoviruses (type 5 and 12) [105,106]. The antiviral activity of *C. majus* against retroviruses has been proven through isolation of the anti-HIV-1 latex compound named ChM-P2 from its aqueous extract [99]. Analysis has shown the low-sulfated poly-glycosaminoglycan character of the isolated molecule. It prevents infection of human CD4^+^ T-cell lines (AA2, H9) with HIV-1 and subsequent cell death. Virus-induced syncytium formation as well as lower cell-to-cell virus spread were also observed in H9 cells. The anti-retroviral activity of the ChM-P2 substance was also confirmed in vivo in mouse AIDS (MAIDS) model C57Bl/6 [99]. The activity of *C. majus* against herpesvirus, influenza virus, and poxviruses has also been tested on albino mice by Lozjuk et al. The effectiveness of *C. majus* alkaloids was estimated on the median duration of mouse life, degree of pulmonary tissue changes and the differences in median HR titres. All of the used alkaloids have shown an inhibiting effect on the infection [107]. *C. majus* latex was also used in oral form to treat a group of 20 patients suffering from SARS-CoV-2 infection with significant clinical improvement after three days of drug administration [86].

Antiviral properties of latex ingredients have been also presented in different plant species. A study conducted by Camero et al. showed the in vitro antiviral activity of fig fruit *F. carica* latex against caprine herpesvirus-1 (Cp-HV1) through a reduction of viral titers produced by MDBK cells [108]. Although the Cp-HV1 affects goats, it shares significant similarities with human genital herpes virus (HHV-2), and therefore it can serve as a model in further research. In another study of *F. carica* latex, presented by Houda Lazreg Aref et al., the hexane and ethyl acetate–hexane latex extracts were proven to be active against herpes simplex virus (HSV-1), european catfish virus (ECV-11), and adenovirus [109]. Such activity can be caused by the presence of ferulic acid being the major phenolic compound in the *F. carica* latex extracts. Lyophilized extracts from *Momordica charantia* L. were also shown to be active against HSV-1 virus, along with their antiviral activity towards sindbis virus (SINV) [110]. Other antiviral agents that have been found in latex of medical plants include inophyllum, coumarins, and calanolide A from *Calophyllum teysmannii* Miq., which serves as a unique non-nucleoside reverse transcriptase inhibitor and can be effective against HIV-1 [111]. Oligomeric proanthocyanidin (SP-303) from *Croton lechleri* latex was shown to inhibit respiratory syncytial virus (RSV) and HSV viral absorption and penetration through the plasma membrane [112]. In turn, (+)-pinoresinol-4-O-β-D-glucopyranoside from *Calotropis gigantea* L. latex presented anti-influenza activity towards a panel of human viruses (A/PR/8/34 (H1N1), A/FM/1/47 (H1N1) and A/Aichi/2/68 (H3N2)) [113].

### 5.2. Cytotoxicity

*C. majus* along with other medical plants are often studied in the context of cancer treatment and cytotoxicity. In numerous studies, cytotoxicity has already been confirmed towards keratinocytes, cells tightly linked to HPV life cycle [35,114,115,116]. Sanguinarine, protopine, and less noticeably chelidonine were able to inhibit the growth of keratinocytes as well as apoptosis of MT-4 cells present in acute T lymphoblastic leukemia. Chelidonine from *C. majus* was found to block the cell cycle of MT-4 cells at G2/M phase. Although it does not bind to DNA directly, it was more successful at apoptosis induction than sanguinarine [117]. Chelidonine was also involved in the apoptosis in HeLA cells by activation of signaling pathways connected to p38–p53 proteins and AKT/PI3 kinase. Morphological analysis has shown the cell shrinkage and blebbing which are typical for apoptosis. Levels of MAPK enzyme p83 responsible for ROS-induced apoptosis in cells were also up-regulated. Chelidonine caused the increase in sub-G1 and G0/G1 cell populations, demonstrating an inhibitory effect. It also led to an increase of mitochondria membrane permeability, thereby allowing for the release of cytochrome c and activation of Apaf-1 involved in apoptosis. The treatment of HeLa cells with *C. majus* extracts also resulted in a decrease of the expression of PI3K, JAK3, AKT, and STAT3 pathways involved in numerous cellular processes, such as apoptosis, survival, proliferation, and cell growth. The down-regulation of oncogenic E6 and E7 HPV proteins was also noticed, further showing its anticancer potential [118]. It was also found to induce expression of telomerase reverse transcriptase (hTERT) and accelerate the senescence of the cells through activation of telomerase in HepG2 cells [117]. In other studies, five alkaloids (sanguinarine, chelidonine, protopine, stylopines) from *C. majus* latex have been tested against melanoma cells, which led to apoptosis of the cancer cells with only a mild effect on normal cells [119]. The effectiveness of *C. majus* milky sap on dermal tumors has been also tested by Isolde Riede’s team. Clinical data have proven the destruction of pathologically altered tissues and eradication of preneoplastic lesions after regular application of the latex [120]. A study conducted by Nawrot et al. has shown the ability of CMN1 and CMN2 nucleases from the milky sap to exert an apoptotic effect after 48 h on neoplastic cell line HeLa with no significant effect on ovarian fibroblast cells of Chinese hamsters. The activity depended on the concentration of nucleases as well as the season time of *C. majus* latex collection. In the case of CMN2, lower pro-apoptotic activity was noticed in October, compared to May. This can be explained by differences in the post-translational modification of proteins as well as by the amount of certain enzyme cofactors [19].

Different plants from the *Ficus* genus have also exhibited cytotoxic activity towards cancer cells. Study conducted by Azza M. Abdel-Aty et al. has shown the cytotoxic activity of phenolic latex extracts from *Ficus sycomorus* L., *F. carica,* and *Euphorbia tirucalli* L. towards the acute myeloid leukemia HL-60, liver HepG2, and breast MCF-7 cancer cell lines. *F. carica* extract exhibited moderate cytotoxic activity towards colon HCT116 cancer cell line and latex extract from *E. tirucalli* had moderate cytotoxic effect on lung A549 cancer cell line. The cytotoxic effect was similar to doxorubicin, which is already used as an anticancer drug. Additional HPLC analysis of latex extracts used in the study helped to distinguish a few bioactive compounds, which include, among others, psoralen, xanthotoxin, phthalic acid, and lanosterol. *F. carica* latex ethanol, dichloromethane, and ethyl acetate extracts were also found moderately cytotoxic towards the HeLa cell line with no significant differences between extracts and crude latex [121]. In a different study conducted by Tulasi et al., the solvent extracts of *Ficus benghalensis* L. and *Ficus religiosa* L. showed an anti-proliferative effect on breast MCF-7 cancer cell line with a 90% inhibition rate at the highest concentration of extract used (200 µg/mL) [122].

Cytotoxic activity towards cancer cell lines has also been proven for other plants from the *Euphorbia* genus. In the study carried out by Lívia E.C.Luz et al., the cytotoxic effect of *Euphorbia umbellata* (Pax) Bruyns latex extract was tested on HeLa and HRT-18 cells. Both cell lines were responsive to treatment and morphological analysis demonstrated the presence of apoptotic events and signs of severe toxicity [123]. *E. umbellata* latex was also used on melanoma cells (B16F10) and cytotoxicity in vitro, as well as in vivo were confirmed. Latex application was responsible for lowering the tumor mass in mice. Such activity was linked to the presence of triterpenes in the plants latex [123]. Cytotoxic activity of *Euphoria* genus latex was also proven by using *Euphorbia helioscopia* L. latex to treat hepatocellular carcinoma in nude mice xenograft. A higher concentration of latex caused the downregulation of cyclin D1 expression, a protein associated with cell cycle regulation (G1 phase), which resulted in cell proliferation inhibition, as well as apoptosis induction. Another protein whose expression was downregulated was bcl-2 protein, known as an antiapoptotic factor. Latex administration, on the other hand, increased the expression of two proapoptotic factors, bax and caspase-3, which induced cell apoptosis in xenografts. A significant decrease of MMP-9 protein, responsible for degradation of the extracellular matrix, was also confirmed. Together with overexpression of nm23-H1 protein, involved in metastasis inhibition processes, it allowed the suppression of cancer cell invasion and migration [124]. Another species from the *Euphorbia* genus, *Euphorbia macroclada* Boiss. was used against breast cancer cell lines (MDA-MB-468 cell line) with Taxol, an anticancer drug, as a positive control. Dichloromethane and ethyl acetate extracts have shown cytotoxic activity, which resulted in at least 50% growth inhibition of cancerous cells [125]. Another in vivo study has been conducted using mice animal model to present *C. procera* dried latex cytotoxic effect on hepatocellular carcinoma cells [126]. For 15 weeks, mice were orally administered with dried latex which resulted in significant decrease of vascular endothelial growth factor (VEGF) levels in the serum. Since VEGF serves as a marker of angiogenesis, the chemopreventive effect of oral administration of *C. procera* latex in vivo can be assumed. An in vitro assay also confirmed the cytotoxic activity via the increased activity of cellular nucleases, which led to DNA fragmentation. Cytotoxic activity in vitro was associated with polar fractions and studies on different cell lines: non-hepatoma (COS-1), hepatoma (Huh-7), and non-cancerous line (AML12). Cytotoxicity was strongly selective for transformed cells.

### 5.3. Antimicrobial Activity

Because of the rising concern related to antibiotic resistant bacteria, it is crucial to look for alternative antimicrobial compounds. Examples of the use of different plant species latex along with treated bacteria and fungi species are presented in Table 3. Studies by Colombo et. al. have shown the quaternary ammonium groups of isoquinoline alkaloids to be responsible for antibacterial activity since both natural and synthetic compounds lacking those groups had no antimicrobial activity [127]. *C. majus* antimicrobial activity has been confirmed in a few studies, for separated compounds which included different alkaloids and glycoproteins summarized in Table 3 [128,129,130,131]. For example, studies conducted by Pavão and Pinto have shown that berberine, coptisine, and sanguinarine had antibacterial effects on *Bacillus subtilis* [132].

The presence of antimicrobial peptides (AMP) has been noted in latex-bearing plants and is also responsible for antimicrobial characteristics. The majority of AMPs consist of short peptide sequences (between 12 and 50 amino acids) with about 50% of amino acids present being hydrophobic (Val, Ile, Phe, Trp, Leu), therefore allowing AMPs to penetrate cell membranes [146]. They have been proven to act against bacteria and exhibit immunomodulatory properties through suppression of the inflammatory response and stimulation of the host’s immune response [147]. They show activity against bacteria, viruses, fungi, and parasites [148,149]. In the study of J. Siritapetawee et al., the activity of 48-kDa protease (AMP48) from *Artocarpus heterophyllus* Lam. latex has been proven to act against *Pseudomonas aeruginosa* and *Candida albicans* (Table 3) [135]. In the case of bacteria, AMP48 was able to alter the morphology of the bacteria cell and therefore significantly reduce its size after the treatment. Other studies have presented the activity of hevein, a small cysteine-rich peptide found in *H. brasiliensis* latex. The inhibition of hyphal growth of fungi by interacting with chitin present in the fungi cell wall was demonstrated [150].

Other examples of antimicrobial latex activity are related to studies on *Aloe harlana,* specifically two latex proteins anthrone (aloin) and chromone (7-O-methylaloeresin A), which were tested against 23 bacterial and four fungi strains (Table 3). The antimicrobial activity of those proteins was comparable to the reference drugs. Additional screenings also showed in vitro antioxidant activity (in 2-deoxyribose and DPPH degradation assays), which is probably caused by the phenolic nature of aloin and chromone [133].

It is also crucial to note that plant latex can serve as a support to traditionally used antibiotics or antimicrobial medicines. For example, *C. procera* latex can act synergistically when used with reference drugs. Both Ciprofloxacin and Clotrimazole had better efficacy when used together with crude latex extract of *C. procera*. The modification of therapeutic doses can help with potential side effects, drug–drug interactions, and the amount of medicine and latex used [137].

### 5.4. Immunomodulatory Properties

Complexes of proteins and polysaccharides from *C. majus* (CN-Ala) have been shown to have immunomodulatory potential. Such complexes had a mitogenic activity on the bone marrow and spleen cells, along with increasing the levels of granulocytes and macrophage colony stimulating factors (GM-CSF) [151]. Another study has demonstrated the immunomodulatory effect of *C. majus* latex using mouse peritoneal macrophages. The combination of latex and recombinant interferon (rIFN-y) led to a significant increase in nitric oxide (NO) production, expression of inducible nitric oxide synthase (iNOS), and increase in TNF-alpha production. It is important to note that the *C. majus* latex acted synergistically with rIFN-y as the combination of two had better effect on NO, iNOS and TNF-alpha production than the use of separate compounds [152]. Extracts isolated from *C. majus* were also able to increase humoral and cell-mediated immunity and reduce the frequency of relapses in pharyngitis in children with chronic tonsillitis [153]. Such immunomodulatory effects are thought to be connected with cytoprotective effects through the alleviation of oxidative stress and reduction of the proinflammatory cytokines levels (such as TNF-α, IL-6) [6]. The study conducted by Danielle Cristina de Oliveira Nasciment et al. examined the immunomodulatory properties of latex from the *C. procera* plant against the experimental infection of *Listeria monocytogenes*. *C. procera* latex up-regulated the pro-inflammatory cytokines, involved in leukocyte recruitment and activation. It also prompted TNF-α and IL-6 mRNA transcripts after infection which resulted in higher efficacy of intracellular bacterial killing. Additional in vivo studies on Swiss mice have shown that even one administration of *C. procera* latex resulted in higher survival rate, ease of symptoms of infection, and accumulation of leukocytes in bloodstream along with its increased migration into the peritoneal cavity [154].

Other immunomodulatory activities include immunosuppression effect through reduction of antibody titer (delayed type hypersensitivity), which can be used in the treatment of liver disorders, enhancement of activity of Th1 and Th2 helper T cells as well as natural killer (NK) cells [155], enhancement of production of serum lysozyme, total serum proteins, as well as tissue superoxide dismutase (SOD) and immunoglobulins [156]. There are also reports of phagocytosis activity and stimulation of humoral immunity [157,158,159,160,161].

Although medicinal plants carry numerous biomedical benefits to human health, it is also worth noting the potential toxicity of plant extracts. The most important is the hepatotoxic activity which can bring undesirable effects when administering the drug orally. Liver damage after consumption of *C. majus* herb extracts have been noticed. After discontinuation of treatment symptoms resolved and the liver recovered within 2 months [97,162,163]. Due to the hepatotoxic effects, which are assumed to be connected to pharmacological interaction with non-steroidal anti-inflammatory drugs or hormones, caution is needed [6]. Another safety issue is related to phototoxicity. When administering the plant-based drug on skin, patients should be alerted to the potential danger of sun exposure. The efficacy of the treatment is dependent on several factors, which include the need for regular application or oral administration of the drugs, time of plant harvest since the concentration of secondary metabolites, and protein content variability between seasons, as well as the individual profile of the patients since some subsequent hypotheses suggest that there is always a certain percentage of patients resistant to treatment, no matter what kind of drug is tested [164]. Because of still unknown mechanisms underlying the mode of action of certain latex biomedical compounds, the possibility of side effects such as liver issues, and the small number of clinical studies, further experiments must take place in order to successfully introduce more medicines from plant latexes to the drug market.

## 6. CRISPR/Cas 9 System as Future Direction for Functional Analysis of Proteins

**Figure 2:**
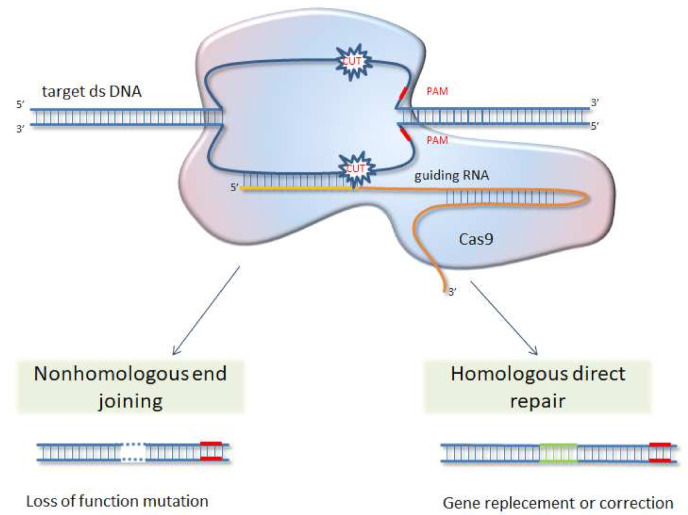
Scheme of CRISPR/Cas9 genome editing technology. Guide RNA, directed by PAM sequences near the targeted gene, lead Cas9 nuclease to altered DNA in desired location. Double strand breaks are repaired either by NHEJ or HDR mechanisms upon the existence of a donor template, which in result lead to deletion or insertion and gene knockout. NHEJ is more efficient than HDR, but may produce indel mutations, whereas HDR can provide a precise gene modification.

**Figure 3:**
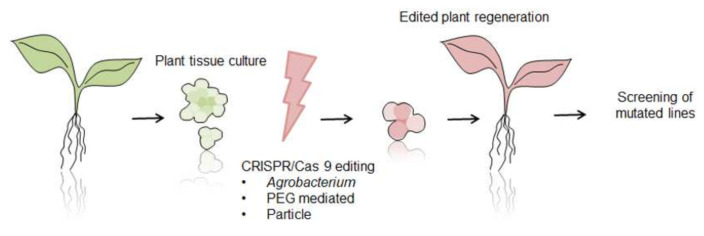
Schematic overview of plant genome editing with CRISPR/Cas9 tool. After obtaining tissue culture from a plant of interest, compounds of CRISPR/Cas9 construct are delivered through *Agrobacterium*, PEG or particle bombardment. In the next step selection of mutated lines, regeneration and screening of mutated plants are performed.

Since 2013, the CRISPR/Cas9 (clustered regularly interspaced short palindromic repeats/CRISPR associated protein 9) genome editing system, with its Cas9 nuclease directed by target-specifying single guide RNA (sgRNA), has emerged as a practical method for the functional analysis of proteins and then as promising tool for the breeding of new varieties [165]. The CRISPR/Cas9 is a naturally occurring mechanism in bacteria and a few Archaea species. It works similarly to human immune systems and enables bacteria to acquire resistance to viruses after infection [166]. Genome editing with the use of CRISPR/Cas9 system is achieved by the induction of site-specific double-strand breaks, which are in turn repaired by either non-homologous end-joining (NHEJ) or homology-directed repair (HDR) machinery of the cell (see Figure 2 and Figure 3). Guide RNAs are designed to identify a three-base-pair protospacer adjacent motif (PAM) sequence occurring downstream of the target DNA. Then DNA is cut by Cas9 nuclease leading to gene knock-out [167]. The CRISPR/Cas9 tool has advantages over previously used methods, like zinc-finger nucleases (ZFNs) and transcription activator-like endonucleases (TALENs), in being easier with simple cloning steps needed, high ease of multiplexing (knockout multiple genes), and large-scale library preparation capacity. This method was successfully used in studies of different gene function, regulatory elements, and genetic mechanisms underlying quantitative trait loci (QTLs) in model organisms, as well as major crop species [168,169,170,171,172,173,174]. Several attempts of laticiferous plant modifications with CRISPR/Cas9 were also taken and are surveyed below.

Due to the high importance of natural rubber used to manufacture about 50,000 products, from tires to medical gloves, the Brazilian rubber tree (*H. brasiliensis*), a main source of rubber on a global scale, is subject to modification with CRISPR/Cas9 technology. Five sgRNAs were introduced to *H. brasiliensis* protoplast culture and targeted five different genes involved in flowering regulation-two genes form *FLOWERING LOCUS T* (FT) subfamily and three genes form *TERMINAL FLOWER1* (TFL1) subfamily. Using the RNP-based genome editing system, modifications were introduced in all five genes, from which majority were deletions. The −1 nt deletion of the fourth nucleotide upstream of the PAM sites was the most frequently observed in all cases [175]. The rubber tree is a perennial tree species with a long juvenile phase, so conventional breeding for agronomic trait improvement is time consuming. The creation of early-flowering or delayed-flowering rubber tree plants will undoubtedly push forward studies related to the improvement of yield and quality, along with disease and stress resistance traits of this plant species.

Another example of laticiferous plant modification is the genetic engineering of *Taraxacum kok-saghyz* L.E.Rodin, commonly named Russian dandelion. It is an undomesticated dandelion species which can be a natural rubber source, alternative to *H. brasiliensis*. CRISPR/Cas9 was deployed to knockout gene encoding *fructan:fructan 1-fructosyltransferase* (1-FFT), a key enzyme in inulin biosynthesis [176]. Inulin is considered the main antagonist of rubber production, so the reduction of its synthesis should boost rubber particles formation. Such a modification not only shed lights on rubber biosynthesis mechanisms, but accelerates the domestication of dandelion as a rubber producing crop [177]. The application of *A. rhizogenes*-mediated hairy root induction allows to quickly obtain plants with a mutation rate as high as 80.0% (in regenerated plants).

For the enhancement of *T. kok-saghyz* agronomic performance, the CRISPR/Cas9 system was also used to induce a mutation in a gene called Rapid Alkalinisation Factor 1 (RALF1). In *A. thaliana,* RALF1 has been shown to suppress root growth [178]. As rubber is extracted from dandelion roots, it should be beneficial to change its morphology from branched to taproots, which are easier to harvest, and the wasted yield of lateral roots is minimalized. Knockout of gene TkRALFL1, achieved using *Agrobacterium tumefaciens*, resulted in introduction of premature stop codon or shortened sequence, which caused the removal of the functionally critical cysteine residues on the protein level. The root volume was 35% higher on average in the heterozygous knockout plants and 60% higher on average in homozygous knockout plants. Moreover, the inulin levels were higher in the knockouts whereas rubber levels were lower. Lower rubber content was compensated for with a higher dry weight of modified roots, such as the total yield per plant of inulin and rubber, which were much higher in knockout plants than in control [179]. It was shown that modification of gene TkRALFL1 can be used in subsequent breeding of profitable new dandelion varieties.

*P. somniferum*, as mentioned before, is a rich source of clinically important metabolites, which belongs to a group of benzylisoquinoline alkaloids (BIAs). For a better understanding of the gene regulation of those compounds synthesis, the CRISPR/Cas9-based gene knockout system was used to alter BIAs biosynthesis pathway. Enzyme 3′-hydroxy-N-methylcoclaurine 4′-O-methyltransferase (4′OMT), which catalyzes the conversion of central intermediate in BIAs synthesis, was targeted in Agrobacterium-mediated transformation. As a result, decreased total alkaloid content was confirmed. Most dramatic reductions were found in S-reticuline and laudanosine, direct products of 4′OMT [180].

For *Cannabis sativa*, a laticiferous plant species with growing importance in human therapies, a stable transformation protocol for modification with CRISPR/Cas9 strategy has been established recently. In the first step, five genes previously recognized as plant development regulators, were cloned to hypocotyls isolated from the DMG278 variety. Agrobacterium-mediated transformation led to the stimulation of shoot induction, most prominent for the combination of two genes (*CsGRF3-C.,* sativa GROWTH-REGULATING FACTOR and *CsGIF1-C.,* sativa GRF-INTERACTING FACTOR). After optimization of variety and explant, the constructs expressing sgRNA targeting the CsPDS1 gene were co-transformed into protoplasts from modified shoot culture. CsPDS1 encode phytoene desaturase (an enzyme essential for plant carotenoid biosynthesis) and is a common marker gene that can test genetic manipulation tools. Successful knockout of phytoene desaturase results in an easily recognized albino phenotype. Finally, four calli generated white seedlings with the edited CsPDS1 gene were obtained (account for 2.48% of the generated shoot) [181]. This is the first report of successful gene editing as well as stable transformation in *C. sativa*, which opens new possibilities to improved cannabis varieties production.

## 7. Conclusions

In the last decades, we have witnessed a huge expansion of demand for herbal medicines, phytonutrients, or nutraceuticals in developing as well as developed countries. They have become a substantial proportion of the global drug market. Moreover, it is estimated that at least four million people rely on herbal medicines as primary sources of healthcare [182]. Therefore, the need for new therapies, but also for plant-derived drugs with a long history of medicinal use (e.g., morphine), is still growing. Laticiferous plant species are a rich source of bioactive compounds (secondary metabolites and proteins), with only partially known medicinal use. The advent of new high throughput sequencing technologies and the fast, efficient genome editing system of CRISPR/Cas9 gives the research community the tools necessary to fulfill the broadened gap between supply and demand for medicines of plant origin. One of the greatest constraints to working with latex-bearing plants is insufficient genome information. Exploration of the genome of the plant of interest enables the precise modification and avoidance of off-target mutations. Nevertheless, the application of CRISPR/Cas9 modification sheds a new light on the function of some genes in laticiferous plant species, as well as sets directions for the improvement of agronomically important traits. Moreover, it provides a modern basis for further exploration and pharmacological utilization of latex compounds.

## Figures and Tables

**Table 2:** Model of antiviral response of *Chelidonium majus* latex based on in vitro studies, which represents preformed immediate defense response with exuding latex. 1st line of defense-after mechanical damage (e.g., herbivore bite) the latex exudes and due its stickiness clots and stops or kills the herbivore. 2nd line of defense-cell wall damage is the prerequisite for the possibility of viral infection. Thereafter it can be stopped by oxidative burst and antiviral response (3rd line of defense). Abbreviations: PPO–polyphenol oxidase; LOX-lipoxygenase; POX-peroxidase; MLP-major latex protein; GRP-glycine-rich protein. According to [74].

Line of Defense	Type of Action	Predominant Proteins and Compounds
1st	Mechanical damage	PPO, LOX (latex stickiness, different chemicals)
2nd	Oxidative burst	POX, LOX et al.
3rd	Antiviral activity	MLP, GRP (RNase/DNase activity, nucleic acid binding)

**Table 3:** Examples of antimicrobial activity of latex bearing plants.

Latex-Bearing Plant Species	Examined Bacteria	Examined Fungi	Bioactive Compounds	Reference
*Aloe harlana* Reynolds	*Bacillus pumilus* (82) *Bacillus subtilis* (ATCC 6633) *Escherichia coli* (CD/99/1, K88, K99, LT37, ROW 7/12, 3:37C, 306, 872) *Salmonella typhi* (Ty2) *Shigella boydii* (D13629) *Shigella dysentery 1* *Shigella dysentery 8* *Shigella flexneri* (Type 6) *Shigella sonnei 1* *Staphylococcus aureus* (ML267) *Vibrio cholerae* (85, 293, 1313, 1315)	*Aspergillus niger* (ATCC 6275) *Candida albicans* (ATCC 10231) *Penicillium funiculosum* (NCTC 287) *Penicillium notatum* (ATCC 11625)	Anthrone (aloin) Chromone (7-O-methylaloeresin A)	[133]
*Aloe weloensis* Sebsebe	*Enterococcus faecalis* *Escherichia coli* *Pseudomonas aeruginosa* *Staphylococcus aureus*	-	Alkaloids Anthraquinone Flavonoids Glycosides Tannins Terpenoids	[134]
*Artocarpus* *heterophyllus* Lam.	*Bacillus subtilis* *Klebsiella Pneumoniae* *Pseudomonas aeruginosa* (ATCC 27853) *Streptococcus haemolyticus* *Salmonella typhi*	*Aspergillus niger* *Candida albicans*	48-kDa protease (AMP48)	[135,136]
*Calotropis procera* (Aiton) W.T.Aiton	*Bacillus cereus* *Bacillus subtilis* *Escherichia coli* *Klebsiella Pneumoniae* *Pseudomonas aeruginosa* *Salmonella typhi* *Staphylococcus aureus* *Staphylococcus epidermidis* *Streptococcus haemolyticus* *Streptococcus pneumoniae*	*Aspergillus flavus* *Aspergillus niger* *Candida albicans* *Candida tropicalis* *Penicillium chrysogenum* *Saccharomyces cereviciae*	-	[137]
*Calotropis gigantea* L.	*Bacillus cereus* *Escherichia coli* *Lactobacillus acidophilus* *Micrococcus luteus* *Staphylococcus aureus* *Streptococcus mutans*	*Candida krusei*	Alkaloids Phenolic Steroids Terpenes Cardiac glycoside	[138,139]
*Carica papaya* L.	*Bacillus subtilis* *Klebsiella Pneumoniae* *Streptococcus haemolyticus* *Salmonella typhi*	*Aspergillus niger* *Candida albicans*	-	[136]
*Chelidonium majus* L.	*Aeromonas hydrophila* *Agrobacterium tumefacians* *Bacillus cereus* *Bacillus subtilis* *Candida albicans* *Escherichia coli* *Micrococcus luteus* *Mycobacterium phlei* *Salmonella enteritidis* *Sarcina lutea* *Staphylococcus aureus*	*Candida albicans*		[127,128,129,130]
*Euphorbia heterophylla* L.	*Bacillus subtilis* *Proteus vulgaris* *Pseudomonas aeruginosa* *Staphylococcus aureus*	*Aspergillus niger* *Fusarium oxysporum* *Penicillium sp.*	Alkaloids Flavonoids Phenols Saponins Steroids Tannins	[140]
*Ficus carica* L.	*Enterobacter cloacae* *Enterococcus faecalis* (ATCC 29212) *Escherichia coli* *Escherichia coli* ATCC 25922 *Pseudomonas aeruginosa* (ATCC 2783, ATCC 27950) *Staphylococcus aureus* *Staphylococcus aureus* (ATCC 25923) *Staphylococcus epidermidis* *Staphylococcus saprophyticus*	-	alpha-Amyrenyl acetate Aristolone Bornanone-3 Lanosta-8 Olean-12-en-3-ol, acetate Urs-12-en-24-oic acid	[141]
*Jatropha gossypifolia* L.	*Pseudomonas aeruginosa (CRPA)* *Staphylococcus aureus (MRSA)*	-	Flavonoids	[142]
*Jatropha multifida* L.	*Pseudomonas aeruginosa (CRPA)* *Staphylococcus aureus (MRSA)*	-	Flavonoids	[142]
*Jatropa carcass* L.	*Bacillus subtilis* *Escherichia coli* *Klebsiella Pneumoniae* *Neisseria gonorrhoea* *Pseudomonas aeruginosa* *Salmonella typhi* *Staphylococcus aureus* *Streptococcus haemolyticus*	*Aspergillus niger* *Candida albicans*	Alkaloid Glycoside Saponin Steroid Tannin	[136,143,144]
*Leptadenia hastata* (Pers.) Decne.	*Klebsiella Pneumoniae* *Staphylococcus aureus* *Staphylococcus aureus* (ATCC 29213) *Salmonella typhi*	-	Alkaloids Flavonoids Glycosides Phenolic Proanthocyanidins Saponins Tannins Triterpene	[145]
*Pergularia daemia* (Forssk.) Chiov.	*Escherichia coli* (ATCC 25922) *Klebsiella pneumoniae* *Pseudomonas aeruginosa* (ATCC 27853) *Staphylococcus aureus* *Staphylococcus aureus* (ATCC 29213) *Salmonella typhi*	-	Alkaloids Flavonoids Phenols Saponins Steroids Tannins Terpenoids	[145]
*Secamone afzelii* (Schult.) K.Schum.	*Escherichia coli* ATCC 25922 *Klebsiella pneumoniae* *Pseudomonas aeruginosa* ATCC 27853 *Secamone afzelii* *Staphylococcus aureus* *Staphylococcus aureus* ATCC 29213 *Salmonella typhi*	-	Alkaloids Cardiac glycosides Saponins Tannins	[145]
*Thevetia peruviana* L.	*Bacillus subtilis* *Klebsiella Pneumoniae* *Streptococcus haemolyticus* *Salmonella typhi*	*Aspergillus niger* *Candida albicans*	-	[136]

## Data Availability

Data is contained within the article.
